# Nanoparticle-Mediated Angiotensin-(1-9) Drug Delivery for the Treatment of Cardiac Hypertrophy

**DOI:** 10.3390/pharmaceutics13060822

**Published:** 2021-06-01

**Authors:** Sabrina Sepúlveda-Rivas, Matías S. Leal, Zully Pedrozo, Marcelo J. Kogan, María Paz Ocaranza, Javier O. Morales

**Affiliations:** 1 Medical Technology School, Faculty of Sciences, Universidad Mayor, Camino la Piramide 5750, Huechuraba, Santiago 8580745, Chile; sabrina.sepulveda@umayor.cl; 2 Center for Bioinformatics and Integrative Biology, Facultad de Ciencias de la Vida, Universidad Andrés Bello, Santiago 8370146, Chile; matileal21@gmail.com; 3 Red Para el Estudio de Enfermedades Cardiopulmonares de Alta Letalidad (REECPAL), Santiago 8380453, Chile; zpedrozo@med.uchile.cl; 4 Advanced Center for Chronic Diseases (ACCDiS), Universidad de Chile & Pontificia Universidad Católica de Chile, Santiago 8380494, Chile; mkogan@ciq.uchile.cl; 5 Programa de Fisiología y Biofísica, Instituto de Ciencias Biomédicas, Facultad de Medicina, Universidad de Chile, Santiago 8380453, Chile; 6 Departamento de Química Farmacológica y Toxicológica, Facultad de Ciencias Químicas y Farmacéuticas, Universidad de Chile, Santiago 8380494, Chile; 7 División de Enfermedades Cardiovasculares, Facultad Medicina, Pontificia Universidad Católica de Chile, Santiago 8330024, Chile; 8 Center of New Drugs for Hypertension, Universidad de Chile & Pontificia Universidad Católica de Chile, Santiago 8380494, Chile; 9 Departamento de Ciencias y Tecnología Farmacéuticas, Facultad de Ciencias Químicas y Farmacéuticas, Universidad de Chile, Santiago 8380494, Chile

**Keywords:** polymeric nanoparticles, gold nanoparticles, angiotensin-(1-9), peptide delivery, cardiac hypertrophy

## Abstract

Ang-(1-9) peptide is a bioactive vasodilator peptide that prevents cardiomyocyte hypertrophy in vitro and in vivo as well as lowers blood pressure and pathological cardiovascular remodeling; however, it has a reduced half-life in circulation, requiring a suitable carrier for its delivery. In this work, hybrid nanoparticles composed of polymeric nanoparticles (pNPs) based on Eudragit^®^ E/Alginate (EE/Alg), and gold nanospheres (AuNS), were developed to evaluate their encapsulation capacity and release of Ang-(1-9) under different experimental conditions. Hybrid pNPs were characterized by dynamic light scattering, zeta potential, transmission and scanning electron microscopy, size distribution, and concentration by nanoparticle tracking analysis. Nanometric pNPs, with good polydispersity index and colloidally stable, produced high association efficiency of Ang-(1-9) and controlled release. Finally, the treatment of neonatal cardiomyocytes in culture with EE/Alg/AuNS 2% + Ang-(1-9) 20% pNPs decreased the area and perimeter, demonstrating efficacy in preventing norepinephrine-induced cardiomyocyte hypertrophy. On the other hand, the incorporation of AuNS did not cause negative effects either on the cytotoxicity or on the association capacity of Ang-(1-9), suggesting that the hybrid carrier EE/Alg/AuNS pNPs could be used for the delivery of Ang-(1-9) in the treatment of cardiovascular hypertrophy.

## 1. Introduction

Cardiovascular disease (CVD) is the leading cause of death worldwide. The most common CVDs are coronary heart disease, vascular brain disease, high blood pressure, peripheral vascular disease, rheumatic heart disease, congenital heart disease, and heart failure [1]. All these diseases induce cardiac remodeling, which is characterized by changes in heart size, shape, and function, as observed in cardiac hypertrophy (CH), which is macroscopically defined as an increase in the thickness of the interventricular wall [2]. At the cellular and biochemical level, a hypertrophic heart is characterized by an increase in the size of cardiomyocytes, expression of silent genes in the adult stage but active in the fetal stage, increased number of sarcomeres, and increased total protein synthesis and contractile proteins [3]. A large number of CH treatment drugs have been developed based on different principles—for example, β-blockers involved in blocking sympathetic tone, thiazidic or thiazid diuretics that produce natriuresis [4,5], antirenins that act as calcium channel blockers, and renin–angiotensin–aldosterone system (RAAS) blockers that act on the renin angiotensin aldosterone axis, angiotensin-converting enzyme (ACE) inhibitors, and angiotensin type 1 receptor blockers (Ang) II (ATR1). However, these drugs have not been shown to be more effective in treating left ventricular hypertrophy [6,7]. In this scenario, bioactive peptides have emerged as promising new drugs for the treatment of many pathologies including CVD because they have shown significant therapeutic efficacy, selectivity, specificity, activity, reduced drug interactions, and low toxicity [8,9,10].

Angiotensin-(1-9) (Ang-(1-9)) is a bioactive peptide from RAAS derived from Ang I proteolysis, and it is a relatively more stable intermediary than Ang-(1-7). Ang-(1-9) is present in human and rat plasma in concentration ranges of 2 to 6 fmol/mL [11,12]. The administration of Ang-(1-9) for 2 weeks after myocardial infarction decreased cardiomyocyte hypertrophy in vivo [13] and in vitro induced by norepinephrine (NE) [13].

In previous reports, this peptide has been administered using adenoviral vectors that express fusion proteins that release Ang-(1-9) in rat H9c2 cardiomyocytes or rabbit primary adult cardiomyocytes, stimulated with angiotensin II, isoproterenol or arg-vasopressin [14] and also by an adeno-associated viral vector by means of a vein injection applied directly immediately after induction of MI in murine models [15]. The anti-hypertrophy efficacy of Ang-(1-9) may be hampered by their three-dimensional structure, hydrophilic/hydrophobic nature, and stability [16]. These factors result in Ang-(1-9) poor bioavailability [17,18], high vulnerability, and short half-life, due to the degradation by enzymes and proteases, either at the site of administration or en route to the site of pharmacological action. To solve this, recently, a thermo-sensitive LipoTherm-AuNC nanosystem was developed to enable NIR-triggered Ang-(1-9) release. This liposomal system allowed the effective release of the Ang-(1-9) peptide with a precise control by thermal stimulation at Tm. Ang-(1-9) was appropriately protected from enzymatic degradation in the nanosystems and revealed a triggered release upon NIR irradiation, releasing its complete payload during the time period of the study [19]. In this context, the development of polymer nanoparticles (pNPs) as carriers represents an alternative over traditional drug delivery methods in terms of efficiency and effectiveness [20,21,22]. These controlled release systems must consist of biocompatible materials, and in this sense, biopolymers have extensive application in the development of these systems [23,24]. Among its advantages is that its formulation methods are highly reproducible, the different types of pNPs allow encapsulating both hydrophilic and hydrophobic peptides and can modify its surface area with different ligands to increase its stability and chemical functionality, allowing modulation of the rate of release of the drug [25].

The pNPs obtained by complex coacervation of polyelectrolytes encapsulate bioactive peptides in the polymer matrix at a molecular level. This offers greater advantages as they improve the pharmacological activity of peptides by increasing their stability, protection from degradation, and controlled release [26]. Thus, alginate/chitosan, dermatan sulfate/chitosan, and Eudragit^®^ L100/55 (a polymethyl methacrylate, PMMA, derivative)/chitosan, are some of the pNPs that have been described for the encapsulation of hydrophilic polypeptides [26,27]. PMMA is a largely used polymer for applications in optical, pneumatic actuation, sensor, analytical separation, and conductive devices [28,29]. In addition, due to important properties such as non-toxicity, lower cost, easy processability, biocompatibility, minimal inflammatory reactions with tissues, and greater resistance to fractures, it is widely used in biomedical applications that involves the preparation of bone cements for drug administration/release and cranioplasty approved by the FDA [30,31]. Meanwhile, alginate is one of the most versatile biopolymers through its ability as thickening, gel formation, stabilization of biodegradability and biocompatibility. It is commonly used in industry as a pharmaceutical excipient, and it is also emerging as an important material for the development of polymeric delivery systems [32,33].

Previously, our working group reported a new delivery system based on complex coacervation of alginate and Eudragit^®^, a cationic amino-derivate poly methacrylate (EE) for delivery of the enzyme model lysozyme (Lys). By controlling the sum of total charges (TC) and charge ratio (CR), we have demonstrated that it is possible to control the size and properties of the surface that result in pNPs that have moderate polydispersity and can incorporate Lys efficiently. In addition, its cytocompatibility characterization showed promising results in cell viability and cytotoxicity, with only the highest concentrations of pNPs having a harmful effect on HeLa cells. In general, our results show the potential of these new EE/Alg pNPs for use in biomedical applications and may lead the way in the future development of coacervation nanocarriers for biological drugs administration [34].

On the other hand, gold NPs (AuNP) are one of the most used nanoparticles for the administration of different types of drugs, including cardioprotective drugs [35]. Its surface can be modified as they can react with amino or thiol functional groups, allowing them to be functionalized with different molecules with biological interest such as drugs, peptides, or polymers [36]. They have been shown to exhibit low toxicity, low immunogenicity, and stability under physiological conditions; in addition to their nanometric size, they have the property of spreading through biological barriers, being able to reach a specific site of interest and accumulate in the site [37]. Spherical AuNPs (also called gold nanospheres, AuNS) have been efficiently encapsulated within polymeric matrices of poly (glycolic-co-DL-lactic acid), PLGA, which may be used in the future in theragnostic applications or as contrast agents in dark field images and computed tomography (CT) [38]. Gold nanorods have great potential to be used as multifunctional theragnostic agents for photothermal therapy (PTT), and by CT imaging, it has been shown that macrophages can be localized both in vitro and in vivo through CT and PTT imaging of inflammatory macrophages in ApoE mice femoral artery restenosis [39]. Since CT imaging offers superior assessment of coronary lesions, a CT contrast medium has been developed by coating 50 nm diameter spherical AuNPs with a collagen-localizing peptide, collagen adhesin (CNA35), which targets myocardial scars. The feasibility of using CNA35-AuNP for vascular imaging and collagen-specific addressing in the myocardial scar was demonstrated. The combined use of a nanomaterial with greater attenuation than traditional CT contrast agents and a specific targeting mechanism for the load of myocardial scar can expand the applications of cardiovascular CT [40].

Considering all this prior research, the aim of this work was to develop a hybrid nanocarrier from combining pNPs and AuNS (hybrid pNPs) for the effective transport of the Ang-(1-9) peptide. In a future perspective, these hybrid nanoparticles could be administered in a localized zone to maximize delivery of the peptide in the cardiac zone. This has been shown to specifically target nanoparticles in vitro in cardiac cells and infarcted heart after intravenous injection in vivo, achieving a drug administration system specifically targeted to the infarcted heart [41].

In this current investigation, the incorporation of AuNS did not cause negative effects on either the cytotoxicity or the association capacity of Ang-(1-9), in turn suggesting that these hybrid carriers, EE/Alg/AuNS pNPs, could be used efficiently in the delivery of the peptide Ang-(1-9) for the treatment of pathologies regulated by this peptide, as is the case of CH. In fact, we demonstrated that treatment with hybrid pNPs EE/Alg/AuNS 2% + Ang-(1-9) 20% achieved a decrease in both the area and perimeter of cultured cardiomyocytes stimulated with norepinephrine (NE), demonstrating that hybrid pNPs were effective in preventing hypertrophy in these cells.

In addition to the role in the localized delivery of these peptides for the treatment of heart pathologies that respond to Ang-(1-9), the inclusion of AuNS in these hybrid nanoparticles could offer the benefit of being monitored by imaging techniques, taking advantage of its light absorption and emission characteristics that can be detected by both UV-visible and fluorescence [42], thus showing possible applications in theragnostic.

## 2. Materials and Methods

### 2.1. Materials

The copolymer Eudragit^®^ E (EE) (dimethylaminoethyl methacrylate, butyl methacrylate, and methyl methacrylate with a ratio of 2:1:1) was donated by Evonik Industries (Germany). Low molecular weight (Mv = 50–190 kDa) alginate (Alg), tetra chloroauric acid (HAuCl_4_), citric acid, phosphate buffer (PBS), bovine fetal serum (FBS), DME medium (DMEM), 199 medium, sodium dodecilsulfate (SDS), and Triton X-100 were purchased in Sigma Chemical Co. (St. Louis, MO, USA). Trifluoroacetic acid (TFA), acetonitrile (ACN), oxygenated water (H_2_O_2_), and propidium iodide (PI) were acquired at Merck (Darmstaddt, Germany). The bi-distilled water used for all experiments was produced in the laboratory using Milli-Q-Directed equipment; Millipore, SAS-67120, (Molsheim, France). The peptide Ang-(1-9) was supplied by GL Biochem Ltd. (Shanghai, China). Norepinephrine was acquired from Merck Millipore (Ireland, Cork). SnakeSkin™ 3.5K MWCO Dialysis Tubing, Hyclone Penicilin–Streptomycin solution (Thermo Scientific™, Waltham, MA, USA). DaKO fluorescence mounting media was purchased from DAKO Corporation (Carpentry, CA, USA). Bovine serum albumin (BSA) was acquired at Winkler Ltd. (Santiago, Chile).

### 2.2. Polymeric Nanoparticle Synthesis and Ang (1-9) Encapsulation

#### 2.2.1. Synthesis of Polymeric Nanoparticles EE/Alg

Polymeric nanoparticles (pNPs) were prepared by complex coacervation mixing aqueous solutions of the polycation Eudragit^®^ E (EE) and the polyanion alginate (Alg). The polyanion was added to the polycation following a previously described procedure under specific conditions of pH 4.8 at 25 °C and vigorous stirring [34]. The amounts and ratio of the mixed polymers were determined by quantifying the charge contributions of each polymer as a function of the mass used and the charge density of the monomer. The charge ratio (CR), as shown in Equation (1), was defined as the ratio of positive and negative charges, while the total charge (TC), as shown in Equation (2), was defined as the sum of the positive and negative charges:

CR = charge_EE_ (µmol)/charge_Alg_ (µmol), (1)

TC = charge_EE_ (µmol) + charge_Alg_ (µmol), (2)

where charge_EE_ is the total amount of positive charges contributed by EE and charge_Alg_ is the total amount of negative charges contributed by Alg, both in µmol [43]. Formulations were prepared by controlling the CR between polycation and polyanion (n^+^/n^−^) between 0.1 and 10, while TC (n^+^ + n^−^) was kept between 2 and 20 µmol. The final CR and TC ratios values are found in Table 1.

#### 2.2.2. Synthesis of Gold Nanospheres

Gold nanospheres (AuNS) were synthesized via chemical synthesis by reducing auric salt (AuCl_4_^−^) with sodium citrate in the presence of heat, which was initially described by Turkevich et al. (1951) [44]. For this, 100 mL of tetra chloroauric acid solution (HAuCl_4_, 1 mM) were added in a 250 mL two-neck round-bottom flask. The balloon was placed on a heating mantle and heated at constant reflux for about 5 to 10 min. Then, a solution of sodium citrate dihydrate (38.8 mM) previously heated by water bath at 50–60 °C was rapidly added through the lateral neck of the balloon. The mixture was left with constant stirring, maintaining reflux for exactly 30 min from the time the citrate was added. The resulting solution was left to cool to room temperature; then, it was centrifugated al 5 °C, and the supernatant was separated. Finally, the NPs obtained were filtered with a PVDF filter (0.45 μm) to remove any other pollutant and stored at 4 °C.

#### 2.2.3. Hybrid pNPs Synthesis and Ang-(1-9) Encapsulation

Hybrid pNPs containing Ang-(1-9) and AuNS were prepared by using the complex coacervation method. For this, solutions of EE and Alg 1% *m*/*v* were mixed, adding the solutions of Alg and AuNS in the appropriate case to a solution of EE containing Ang-(1-9) under controlled conditions of pH 4.8 and temperature of 25 °C, applying vigorous agitation of 600 rpm for 25 min. pH 4.8 is the pH of the mixture of polymers when forming the pNPs, and this pH was controlled after adding the AuNS and Ang-(1-9), being maintained almost constant; however, drops of HCl 0.01 M were added to correct for these small variations when required. AuNS values and the Ang-(1-9) concentration values are found in Table 2 and Table 3, respectively.

### 2.3. Physicochemical Characterization

The physicochemical properties of EE/Alg, AuNS and hybrid pNPs such as size, size distribution, and polydispersity index (PDI) were determined by Dynamic Light Scattering (DLS), while the Z-potential (ZP) was determined by Laser Doppler Electrophoresis. Both techniques were performed in triplicate, at 25 °C, using a Zetasizer Nano ZS (Malvern Instruments Ltd., Worcestershire, UK) equipment.

#### 2.3.1. Morphological Characterization

pNP morphology was characterized by scanning electron microscopy (SEM). For this, 1 mL of the selected pNP samples were flash frozen on liquid nitrogen and then lyophilized at −50 °C under high vacuum (0.002 mbar) during 24 h in a Freezone 1 (Labconco, Kansas City, MO, USA). Trehalose 5% *w*/*v* in a 1:3 ratio was used as cryoprotectant. Then, lyophilized powders were coated with a thin layer (10–12 nm of thickness) of Au/Pt in a high vacuum evaporator (108 Auto, Cressington Scientific Instruments Ltd., Watford, United Kingdom) and were analyzed in a SEM Inspector F50 (FEI, Hillsboro, OR, USA) operated at 5 kV.

#### 2.3.2. Molecular Absorption Spectrophotometry

To observe the characteristic surface plasmonic absorption bands of AuNS and their correlation with size, a characterization was carried out using UV-Visible absorption spectrophotometry. The AuNS UV-Vis absorption spectrum was obtained at room temperature, with a Perkin Elmer spectrometer model Lambda 25 UV/Vis. A sweep was performed between 400 and 800 nm, using a 1 cm optical pass disposable plastic cuvette and using sodium citrate 1.2 mM as blank on the double-beam spectrophotometer.

#### 2.3.3. Transmission Electron Microscopy

To study the morphology and homogeneity of AuNS size, a scanning electron microscope with FEI Inspect F50 electronic transmission module (STEM) was used using electron acceleration from 120 keV. The samples were prepared by depositing a drop of an NP suspension (17 μL) on 200 mesh copper grids coated with formarv/coal polymer and allowed to dry for at least 30 min. The excess was removed with filter paper, and 1 drop of phosphotungstic acid was added 1% at 1 min and left drying 24 h at room temperature. To obtain population data, 100 particles were measured using the free ImageJ software representing the data as frequency versus aspect ratio histograms.

#### 2.3.4. Nanoparticle Tracking Analysis

To perform the nanoparticle tracking analysis (NTA), the samples were diluted 1:100 at an appropriate concentration using filtered bi-distilled water prior to analysis. This water was also analyzed for use as a control. The analysis was carried out on an NS300 NanoSight (Malvern Instruments Ltd., Worcestershire, UK) team. All measurements were made at 25 °C and per triplicate for each sample, resulting in video captures per sample at each flow rate. The scattered light from the particles was captured by an sCMOS camera. The average size, concentration, and standard deviation values obtained by the NTA software correspond to the arithmetic values calculated with all particles analyzed by the software.

#### 2.3.5. Determination of Ang-(1-9) Association Efficiency in pNPs EE/Alg

Association efficiency (AE) was indirectly determined by quantifying Ang-(1-9) in the supernatant after centrifugation separation of pNPs and non-encapsulated Ang-(1-9) (13,000 rpm, 20 °C for 30 min). The following equation was used to determine AE:

AE (%) = [(initial mass − supernatant mass)/initial mass] × 100.(3)

The content of Ang-(1-9) on pNPs was quantified by a validated reverse-phase HPLC (RP-HPLC) method on an HPLC (Flexar Perkin Elmer, USA) computer, using a Symmetry 300 C18 column (3.5 μ 4.6 × 150 mm). A known weight of dry solid was dissolved in sufficient pH 7.0 phosphate buffer, an aliquot of 500 L was filtered and eventually stored in vials for HPLC quantification. The mobile phase for Ang-(1-9) consisted of a gradient composed of solvent A (0.1% *v*/*v* of TFA in 100% ACN) and solvent B (0.1% TFA in 100% Milli-Q water). The mobile phase described initially consisted of 90% solvent B and 10% solvent A; then, it consisted of 70% solvent A and 30% solvent B for 15 min before rising again to 100% solvent A for 20 min. The flow was adjusted to 1 mL/min, the temperature remained constant at 25 °C, and the injection volume was 50 L. The quantification limit was determined at 12.5 g/mL at 220 nm. Retention time was 13 min. The HPLC method was validated against linearity, repeatability, quantification limit, and detection limit.

### 2.4. Colloidal Stability of Hybrid pNPs in Different Media of Relevance

The variation in the physicochemical properties of hybrid pNPs was studied when coming into contact and interacting with components present in different media such as PBS pH 7.4, DMEM supplemented with 10% FBS and plasma, incubated at temperature 25 °C and 37 °C for times of 0, 15, 60, and 120 min. For this purpose, newly manufactured EE/Alg/AuNS/Ang-(1-9) hybrid pNPs were diluted in Milli-Q water; from this dilution, 1 mL was taken and incubated in 500 µL of the different means and conditions described to finally obtain a concentration of 34.4 g/mL corresponding to the maximum evaluated concentration of hybrid pNPs with a good safety profile. Hybrid pNPs were sonicated for 25 min and average size, ZP, and PDI were determined for all triplicate experiments, using ultrapure water as negative control.

### 2.5. In Vitro Release Study of Ang-(1-9) from pNPs and Hybrid pNPs

For Ang-(1-9) release studies from EE/Alg pNPs, EE/Alg/Alg-(1-9) hybrid pNPs were deposited in dialysis bags with molecular cutting weight of 3500 g/mol. This bag was arranged inside a 50 mL volume disposable polycarbonate bucket containing a volume of 30 mL of PBS pH 7.4 placed in a thermoregulated bath at 37 °C and 100 rpm. Aliquots were extracted from 1 mL every 1 h until after 7 h and then at 24 and 48 h. It eventually was quantified by HPLC in a Flexar, Perkin Elmer. To determine whether the concentration of AuNS influences the Ang-(1-9) release profile, hybrid EE/Alg pNPs systems with 20% incorporation of Ang-(1-9) were formulated in a fixed manner, and the percentage of incorporation of the AuNS was varied to 2 and 20% of the mass of the solids.

### 2.6. In Vitro Safety Determination of Hybrid pNPs EE/Alg/AuNS/Ang-(1-9)

The experiments were performed in agreement with the Guide for the Care and Use of Laboratory Animals published by the US National Institutes of Health (2016) and were approved by the Institutional Ethics Review Committees from Universidad de Chile. Adult male and 1–3-day-old Sprague–Dawley rats were obtained from the Animal Breeding Facility of the Faculty of Chemical and Pharmaceutical Sciences, Universidad de Chile. Neonatal rat ventricular myocytes (NRVM) were isolated as described by Foncea et al. (1997) [45] Cells were pre-plated for 2 h in plastic Petri dishes to discard non-myocyte cells. The cardiomyocyte-enriched fraction was plated on gelatin-precoated 35 mm plates and grown in DMEM/M199 (4:1) medium containing 10% (*w*/*v*) fetal bovine serum (FBS) and bromodeoxyuridine (100 mM) for 24 h before the experiments.

Safety tests of pNPs were performed in NRVM maintained in high-glucose DMEM medium supplemented with 10% FBS, 100 U/mL penicillin, and 100 μg/mL streptomycin in a 95% air and 5% CO_2_ atmosphere at 37 °C. For the isolation and cultivation of NRVMs, neonate rat hearts were removed and washed in sterile Hank’s medium at 37 °C. The atria were removed, and the ventricles were mechanically disaggregated to undergo successive enzymatic digestions with type II collagenase (0.02 g/100 mL Hank’s) and pancreatin (0.06 g/100 mL Hank’s). To obtain a fraction enriched in NRVM, the differential adhesion to plastic surfaces exhibited by the two cell types present in the heart (NRVM and fibroblasts) was used. The product of the enzymatic digestion was pre-plated on 100 mm plates for 2 h at 37 °C in DMEM: M199 (4:1) (Maintenance medium, MM) 10% FBS. After that time, the cells were harvested, centrifuged at 1000 rpm for 5 min, and resuspended in 20 mL of MM 5% FBS and 10% FCS. The NRVMs were sowed in MM 5% FBS-10% FCS at different densities, according to the experimental needs, in the presence of 100 μM 5-bromo-2′-deoxyuridine, on culture plates previously covered with a gelatine monolayer (2% *w*/*v*). The cells were kept in thermoregulated incubators at 37 °C in a humidified atmosphere with 5% CO_2_ and 95% air.

#### 2.6.1. MTS Assay

The NRVMs in culture (20 × 10^3^ cells/well) were treated with different stimuli, for which they were diluted in the culture medium in the presence of pNPs. DMEM/10% FBS were used as life control, 10% SDS was used as death control, and pNPs were used as vehicle (Milli-Q water) at 37 °C and 5% CO_2_. The assay was performed in triplicate in 96-well plates at set times and then analyzed with a microplate reader (BioTek’s Synergy, Mx) at 450 and 490 nm. Cell viability was calculated by comparing samples with cells incubated in normal culture medium with a survival rate of 100% (life control). Each sample was measured in triplicate, and averages and standard deviation were obtained from three independent experiments.

#### 2.6.2. Cell Viability by Flow Cytometry

NRVMs (3 × 10^6^ per well) were treated at different times (24, 48, and 72 h) with Ang-(1-9) at concentrations of 1, 10, 100 nM, and 1 μM. H_2_O_2_ 100 μM was used as a positive death control for 24 h. The percentage of viability of the cells was determined by determining the incorporation of propidium iodide (PI) into the cells by flow cytometry.

### 2.7. Efficacy Tests in Neonatal Rat Cardiomyocyte

#### 2.7.1. Hypertrophic Stimulus

Experiments were conducted to test the preventative ability of hybrid pNPs on cardiac hypertrophy. First, the NRVM in culture (0.25 × 10^6^ per well) were serum starved for 24 h and were used as a control. Subsequently, they were stimulated with 10 μM norepinephrine for 24 h and treated with the Ang-(1-9) peptide, empty EE/Alg pNPs, and hybrid EE/Alg/AuNS 2% + Ang-(1-9) 20% pNPs during 24 h to evaluate its effects on NRVM. To evaluate the effect of hybrid pNPs on the prevention of the development of NRVM hypertrophy, prior to stimulation with NE for 24 h, cells were incubated with empty EE/Alg pNPs, EE/Alg/AuNS 2% + Ang-(1-9) 20% pNPs, and Ang-(1-9) 10 µM, which was used as a positive control for the prevention of hypertrophy.

#### 2.7.2. Determination of Area, Perimeter, and Sarcomerization of Cardiomyocytes

NRVMs were sown on 12-well plates with 18 mm object covers at a confluence of 80%. After subjecting them to the desired experimental conditions, the cells were washed 2 times with PBS at 4 °C, fixed with PBS 4% paraformaldehyde for 30 min, and permeabilized with PBS 0.1% Triton X-100 for 10 min. Subsequently, the cells were blocked with PBS 1% BSA for 30 min and dyed with rhodamine-phalloidine 1:500 and Hoescht (1:1000) for 1 h. The assembly was carried out on slides using DAKO mounting media. The images were obtained using Zeiss reverse epifluorescence microscope, Axio vert. A1 with hummingbird lighting, and 63× magnification images and were processed with Image J free software.

### 2.8. Sample Size and Statistical Analysis

The results were expressed as an average ± standard deviation. Three separate experiments were conducted for each condition. Comparisons between experimental groups were made with the GraphPad Prism version 6 statistical program (La Jolla, CA, USA), using one-way ANOVA followed by Tukey, Dunnett, or Bonferroni post-analysis. The values of *p* < 0.05 (*), <0.01 (**), and <0.001 (***) were considered statistically significant. For NPs and safety physicochemical characterization experiments, a 20% difference capacity was projected with a conventional standard deviation from the 10% technique and for a value of *p* = 0.05. For the efficacy study in NRVM, a 25% difference capacity was projected with a conventional standard deviation of the technique of 10% and for a value of *p* = 0.05.

## 3. Results

### 3.1. Physicochemical Characterization

#### 3.1.1. Characterization of EE/Alg pNPs

Physicochemical properties such as size, polydispersity (PDI), and Z potential (ZP) of EE/Alg pNPs prepared by complex coacervation were characterized. The formulations made had electrical charge ratios between polycation and polyanion (n^+^/n^−^) between 0.1 and 10. To control the total number of charges, the sum of charges n^+^ and n^−^ was kept at 15 µmol. The results for size, PDI, and ZP are shown in Table 1.

**Figure 1:**
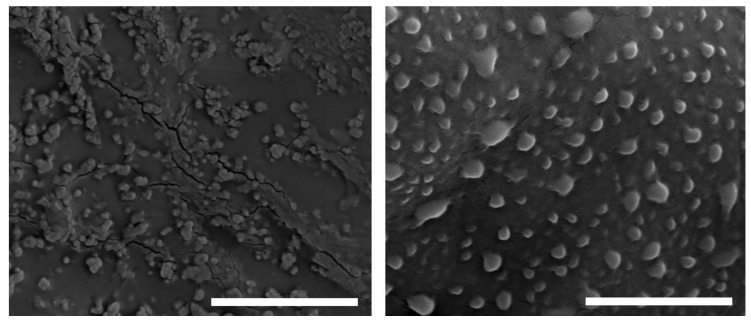
SEM images of EE/Alg (CR = 0.5; TC = 15) pNPs obtained at 30,000× and 60,000×, bar represents 4 μm and 2 μm, respectively.

The images obtained by SEM for the EE/Alg pNPs (CR = 0.5; TC = 15) are shown in Figure 1.

It was observed that spherical particles were obtained in the EE/Alg pNP formulations. The average size measured in the images was 170.7 ± 5.8 nm. When comparing these sizes obtained by SEM and those obtained by DLS (150 ± 5.6 nm), it was found that the nanoparticle sizes measured by both techniques have similar values.

#### 3.1.2. Characterization of Gold Nanospheres (AuNS)

The formation of AuNS is visually confirmed by obtaining a characteristic dark reddish solution that has been described in the literature [46]. The atomic absorption spectrum (Figure S1a) presented an absorption maximum at 519 nm that corresponds to the characteristic resonance plasmon band for AuNS with sizes between 10 and 20 nm. In addition, the value obtained by ZP is −46.2 ± 2.4 mV, which is consistent with that reported in the literature for this type of NP. The negative nature of this charge is due to the addition of citrate as a gold reducing agent in the manufacturing process. These molecules are adsorbed on the surface of NP, counteracting the positive surface charges of the gold atoms present [47]. Using DLS, it was determined that the average size of the AuNS is 12.4 ± 1.7 nm and it has a PDI of 0.213, which indicates a moderate size distribution. These results were confirmed by conducting a population study of 300 NPs observed in the micrographs obtained by TEM. It was determined that they have an average size of 12 ± 1 nm and a size distribution as seen in the image insert (Figure S1b).

### 3.2. Encapsulation AuNS and Angiotensin-(1-9) in pNPs EE/Alg

The incorporation of AuNS in EE/Alg pNPs via the complex coacervation technique was determined by the change in its properties with respect to empty EE/Alg pNPs. For this, empty EE/Alg pNPs were evaluated with AuNS inclusion with different CRs and keeping TC at 10 µmol. These AuNS that are about 12 nm in size are negatively charged. The properties determined for these systems are shown in Table 2.

**Figure 2:**
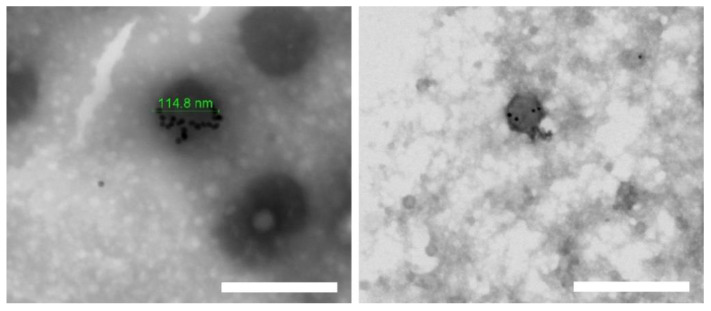
Transmission electron microscopy images of EE/Alg pNPs loaded with AuNS (TC = 15: CR = 1). Bars represent 200 nm (**left**) and 400 nm (**right**).

To obtain the TEM images shown in Figure 2, one of the EE/Alg/AuNS pNP formulations was selected, as marked in Table 2. The criterion used for its selection was based on the size in the nanometric range obtained and its monodisperse distribution [48]. In the TEM micrographs, as shown in Figure 2, an efficient incorporation of AuNS was observed in the matrix of the pNPs EE/Alg. In addition, it was found that when AuNS are incorporated, they do not affect the spherical morphology of pNPs.

On the other hand, EE/Alg pNPs loaded with Ang-(1-9) 2% were evaluated in different CR and keeping the TC at 15 µmol. The values obtained are shown in Table 3.

The characterization by DLS shows that the incorporation of Ang-(1-9) to EE/Alg pNPs with different CR generates pNPs with similar size and PDI, the latter in all cases is less than 0.3. The increase in solids content from CR 0.25 to 10 did not result in an increase in the size of the pNP when incorporating the peptide.

From the characterization study of EE/Alg pNPs loaded with Ang-(1-9) and the study with incorporation of AuNS, the EE/Alg pNPs loaded with Ang-(1-9) were selected at 2% CR 0.5 and 10. Similarly, nanoparticles with percentages of incorporation of Ang-(1-9) of 20% and 40% were characterized, with a negative charge, a positive charge CR of 15 µmol; the results are shown in Table 4.

The three formulations of pNPs EE/Alg/AuNS 2% with 2, 20, and 40% incorporation of Ang-(1-9), as shown in Table 4, presented sizes in the nanometric range. Contrary to what was reported by Umerska et al. (2014) [49], the increase of the Ang-(1-9) peptide did not increase the size of the nanoparticle. This may be due to the attractive forces established between the peptide and its polycation–polyanion, as well as the fact that both EE and Alg do not exhibit a strong cationic–anionic charge. Regarding the ZP, this was positive or negative depending on the CR. Thus, for CR 0.5 a negative ZP was always obtained, since the negatively charged Alg predominated, while for CR 10, the ZP was positive due to the predominance of the positively charged EE. The PDI remained within acceptable ranges for the 2% EE/Alg/AuNS pNPs formulations with 2 and 20% Ang-(1-9). For 2% EE/Alg/AuNS pNPs with 40% Ang-(1-9), PDI ranges of 0.4 were obtained and thus classified as moderate size dispersion.

**Figure 3:**
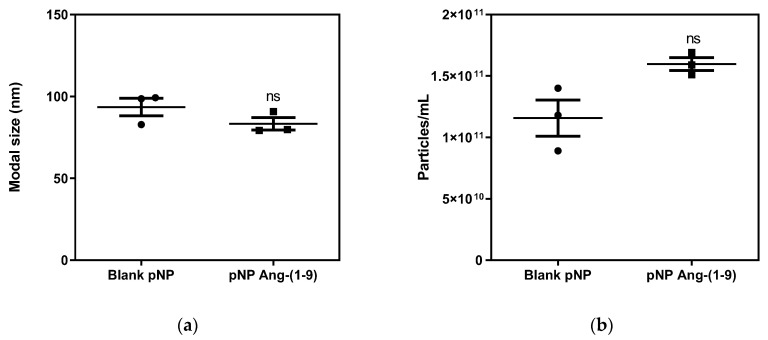
Graphs of (**a**) size and (**b**) concentration of empty EE/Alg pNPs and 2% EE/Alg/AuNS hybrid pNPs + 20% Ang-(1-9). The data represent the mean ± SD (*n* = 3), post-student t-test ns = not statistically significant with respect to empty pNPs.

On the other hand, the results obtained for the distribution of size and concentration of nanoparticles per mL using NTA for hybrid pNPs EE/Alg/AuNS 2% + Ang-(1-9) 20% CR of 0.5 and the empty EE/Alg pNPs are shown in Figure 3.

The concentration of empty EE/Alg pNPs was 1.15 × 10^11^ particles/mL, while for hybrid EE/Alg/AuNS 2% + Ang-(1-9) 20% pNPs, it was 1.6 × 10^11^ particles/mL. The sizes determined by NTA are 95.5 ± 9.3 and 83.2 ± 6.5 nm, respectively; these values are smaller than those found by DLS (150.0 ± 1.1 and 98.1 ± 4.4), but they are still proportional to these values. This difference between the techniques is generally attributed to the amount of statistical data using the DLS technique, which is confirmed by comparing their values in the SD, which are quite high for the NTA.

#### Determination of the Association Efficiency of Angiotensin-(1-9) in EE/Alg and EE/Alg/AuNS pNPs

**Figure 4:**
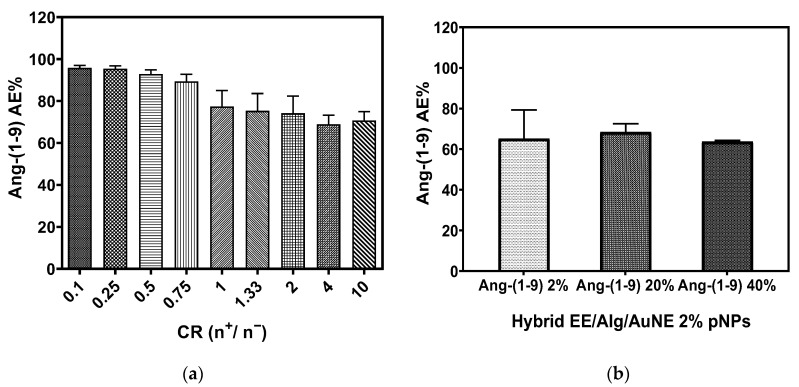
Efficiency of association of (**a**) Ang-(1-9) 20% in EE/Alg pNPs at different CR; (**b**) Ang-(1-9) in 2% EE/Alg/AuNS pNPs. The results were expressed in % EA, which is represented as mean ± SD (*n* = 3).

The association efficiency (AE%) of the Ang-(1-9) peptide was indirectly analyzed by the difference between the theoretical total amount of Ang-(1-9) loaded in the pNPs formulations and the free Ang-(1-9) found in the supernatant. For this purpose, EE/Alg pNPs with Ang-(1-9) 20% were used, and they were evaluated at different CR while TC was set at 15 µmol. A formulation with high association efficiency of Ang-(1-9) and at the same time with a suitable ZP was selected. The results are shown in Figure 4a. It was observed that all the formulations presented a high percentage of association efficiency ranging from 68–95%. In addition, the AE% of Ang-(1-9) was evaluated in pNPs of EE/Alg/AuNS 2% + Ang-(1-9) at 2, 10, and 20%; the results are shown in Figure 4b.

### 3.3. Colloidal Stability of Hybrid EE/Alg/AuNS/Ang-(1-9) pNPs in Different Relevant Media

**Figure 5:**
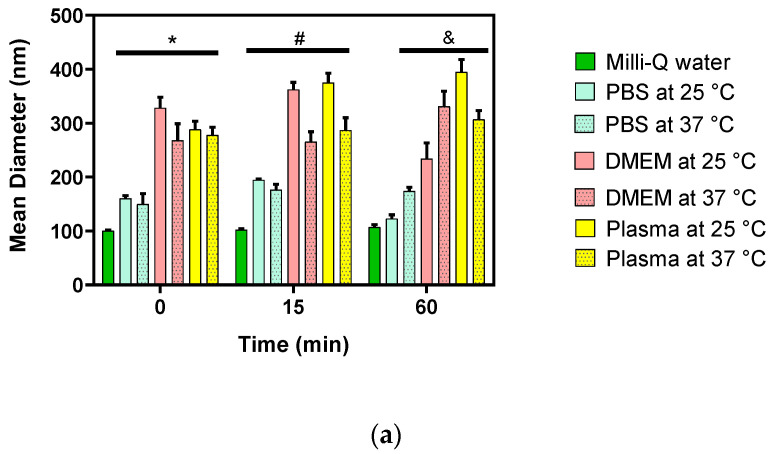

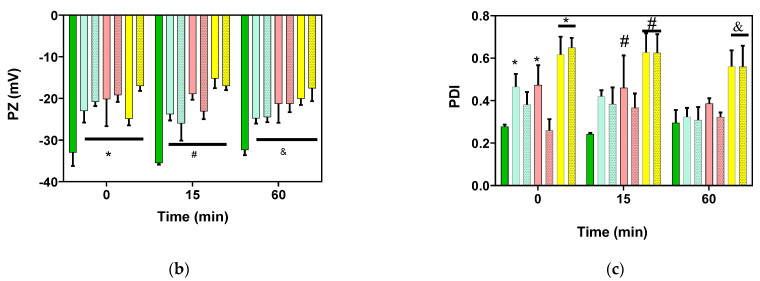
Stability parameters of the hybrid (**a**) mean diameter; (**b**) zeta potential; and (**c**) polydispersity index of pNPs at 25 °C and 37 °C in media (phosphate buffer pH 7.4 (PBS), DMEM supplemented with 10% FBS and plasma and at 0, 15, and 60 min. Milli-Q water was used as a control. The values correspond to the mean DS (*n* = 3). Two-way ANOVA, Bonferroni post-test, * *p* < 0.05 v/s nanohybrids in Milli-Q water at 0 min, # *p* < 0.05 nanohybrid in Milli-Q water at 15 min, & *p* < 0.05 nanohybrids in Milli-Q water at 60 min.

To understand the effects of nanoformulations in vitro, physicochemical parameters must be considered when they interact with biological systems to avoid problems with reproducibility and scaling related to insufficient characterization of the formulations [50]. The hybrid pNPs were characterized by measuring the particle size, ZP, and PDI in different media, such as PBS, DMEM supplemented with 10% FBS, and plasma, comparing with the control of hybrid pNPs in Milli-Q water to study their colloidal stability; see Figure 5.

### 3.4. In Vitro Release Studies of Ang-(1-9)

**Figure 6:**
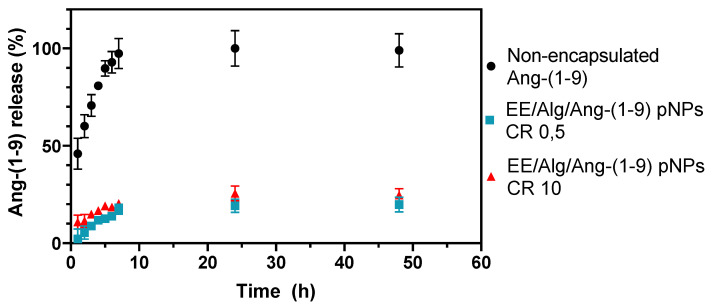
Release profile of non-encapsulated Ang-(1-9) and Ang-(1-9) from pNPs EE/Alg CR: 0.5 and 10, in a dialysis system suspended in PBS pH 7.4 and 37 °C in time intervals. Values are expressed as mean ± SD (*n* = 3).

To determine the release of Ang-(1-9) from the EE/Alg/Ang-(1-9) 2% pNPs and CR of 0.5 and 10, intervals of 1–7 h were used and subsequently measured at 24 and 48 h. A non-encapsulated Ang-(1-9) release profile control was made from the dialysis membrane used in these experiments and reached a complete release during the first 7 h. Both experiments were carried out in PBS at pH 7.4 and 37 °C; see Figure 6.

**Figure 7:**
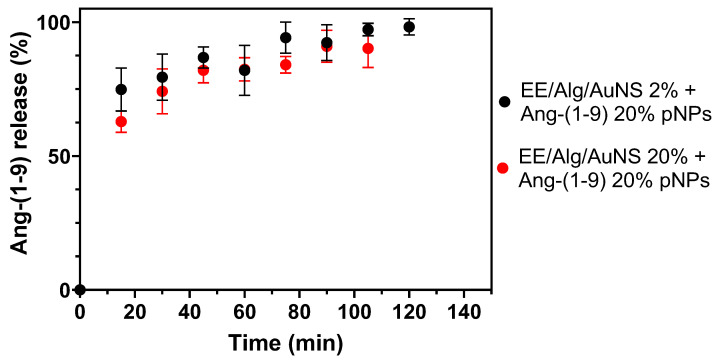
Release profile of Ang-(1-9) from EE/Alg/AuNS 2 and 20% pNPs CR: 0.5, in a dialysis system suspended in PBS pH 7.4 and 37 °C at intervals of time. Values are expressed as mean ± SD (*n* = 3).

On the other hand, Figure 7 shows the release kinetics of the Ang-(1-9) peptide from hybrid EE/Alg pNPs with AuNS. To determine whether the concentration of AuNS influences the Ang-(1-9) release profile, hybrid EE/Alg pNPs systems with 20% incorporation of Ang-(1-9) were formulated in a fixed manner and the percentage of incorporation of the AuNS was varied to 2 and 20% of the mass of the solids.

### 3.5. In Vitro Determination of Safety and Efficacy after Administration of Hybrid EE/Alg/AuNS/Ang-(1-9) pNPs to NRVM in Culture

The use of pNPs in the field of biomedicine has emerged as a possible therapeutic and/or diagnostic tool for certain pathologies such as cardiovascular diseases. To determine their possible toxic effects, NRVM in culture were stimulated with hybrid EE/Alg/AuNS/Ang-(1-9) pNPs at different times (24, 48, and 72 h) and at concentrations of 1, 10, 100 nM, and 1 μM of Ang-(1-9). Figure S2 shows the representative histograms of the distribution of the cell population with respect to its fluorescence by the incorporation of PI, as determined by flow cytometry. It was found that the Ang-(1-9) peptide in its different concentrations does not affect the viability of the treated cardiomyocytes over time. As a positive control for death, 100 μM H_2_O_2_ was used for 24 h. Figure S2 shows the representative histograms for the distribution of the population of cells regarding their fluorescence due to the incorporation of PI, as determined by flow cytometry. In Figure S2B–D, the quantification of the percentage of cells that incorporated PI is presented. Exposure to Ang-(1-9) does not produce changes in cardiomyocyte viability at any of the concentrations or exposure times evaluated. The cell viability assays of EE/Alg/AuNS 2% + Ang-(1-9) 20% pNPs with CR: 0.5 and CT: 15 shown in Figure S3 also did not show significant differences at the different concentrations evaluated of pNPs.

#### In Vitro Anti-Hypertrophic Efficacy of Hybrid EE/Alg/AuNS 2% + Ang-(1-9) 20% pNPs on NRVM Subjected to Stimuli with Norepinephrine (NE)

Considering that cardiac hypertrophy is one of the main pathologies that lead to the development of heart failure and that Ang-(1-9) has been reported to have an anti-hypertrophic role, we evaluate whether this capacity is maintained when this peptide is found encapsulated in our hybrid pNPs. To do this, we measured both the area and the perimeter of NRVMs in culture treated with NE 10 μM for 24 h. Experiments were conducted to test both the preventive and reversal ability of hybrid pNPs on cardiac hypertrophy. For this, the NRVM was treated with the Ang-(1-9) peptide, blank EE/Alg pNPs, and hybrid EE/Alg/AuNS pNPs 2% + Ang-(1-9) 20% for 24 h.

**Figure 8:**
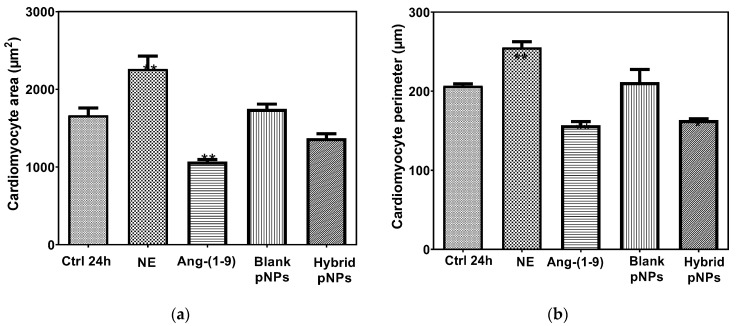
Effect on the area (**a**) and perimeter (**b**) of cardiomyocytes treatment with NE, Ang-(1-9), and hybrid pNPs. Ctrl 24 h: Control cardiomyocytes in their maintenance medium for 24 h, NE: cardiomyocytes + subsequent NE 10 µM, Ang-(1-9): cardiomyocytes + subsequent Ang-(1-9) 10 µM; Blank pNPs: cardiomyocytes + subsequent EE/Alg pNPs, Hybrid pNPs: cardiomyocytes + EE/Alg/AuNS/Ang-(1-9) pNPs. Data represent mean values ± SD (*n* = 3). An ANOVA–Dunnett was performed to compare the 24 h control with the different control treatments, PRISMA Graph * (*p* < 0.05), ** (*p* < 0.001).

The area quantification of the NRVMs is shown in Figure 8a. While the treatment with norepinephrine (NE) increased the area of the NRVM with respect to its control (Ctrl) from 1663.9 ± 219.5 to 2299.9 ± 349.5 µm^2^, the subsequent treatment with the Ang-(1-9) peptide not encapsulated significantly reduced it to 1040.6 ± 58.8 µm^2^. On the other hand, the treatment of NRVM with EE/Alg pNPs and EE/Alg/AuNS 2% + Ang-(1-9) 20%, pNPs both with CR 0.5 and TC 15, showed areas of 1747.3 ± 144.2 and 1370.4 ± 133.4 µm^2^, respectively. Figure 8b shows the graph of the quantification of the perimeter of the cardiomyocytes. As with the data obtained with the area, while the NE significantly increased the perimeter of the NRVMs with respect to its control from 206.7 ± 9.2 to 255.3 ± 18.0 µm, the treatment with the non-encapsulated Ang-(1-9) peptide reduced it to 156.8 ± 9.2 µm, as did the treatment with EE/Alg/AuNS 2% + Ang-(1-9) 20% pNPs to 163.6 ± 5.7 µm.

**Figure 9:**
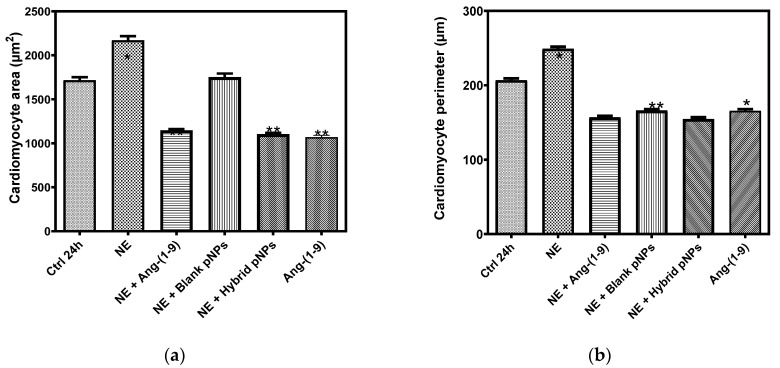
Effect on the cardiomyocyte area (**a**) and perimeter (**b**) of the pre-incubation of different stimuli Ctrl 24 h: cardiomyocyte control in MM (maintenance medium) 24 h; NE: cardiomyocytes 24h + norepinephrine 10 μM; NE + Ang-(1-9): norepinephrine 10 μM + angiotensin-(1-9) 10 μM; NE + Blank pNPs: norepinephrine 10 μM + EE/Alg/Ang-(1-9) pNPs; NE + Hybrid pNPs: norepinephrine 10 μM + pNPs EE/Alg/AuNS/Ang-(1-9)/; Ang-(1-9): cardiomyocytes 24 h + Ang-(1-9) 10 μM. Data represent mean values ± SD (*n* = 3). An ANOVA–Dunnett was performed to compare the 24 h control with the different control treatments, PRISMA Graph * (*p* < 0.05), ** (*p* < 0.001).

**Figure 10:**
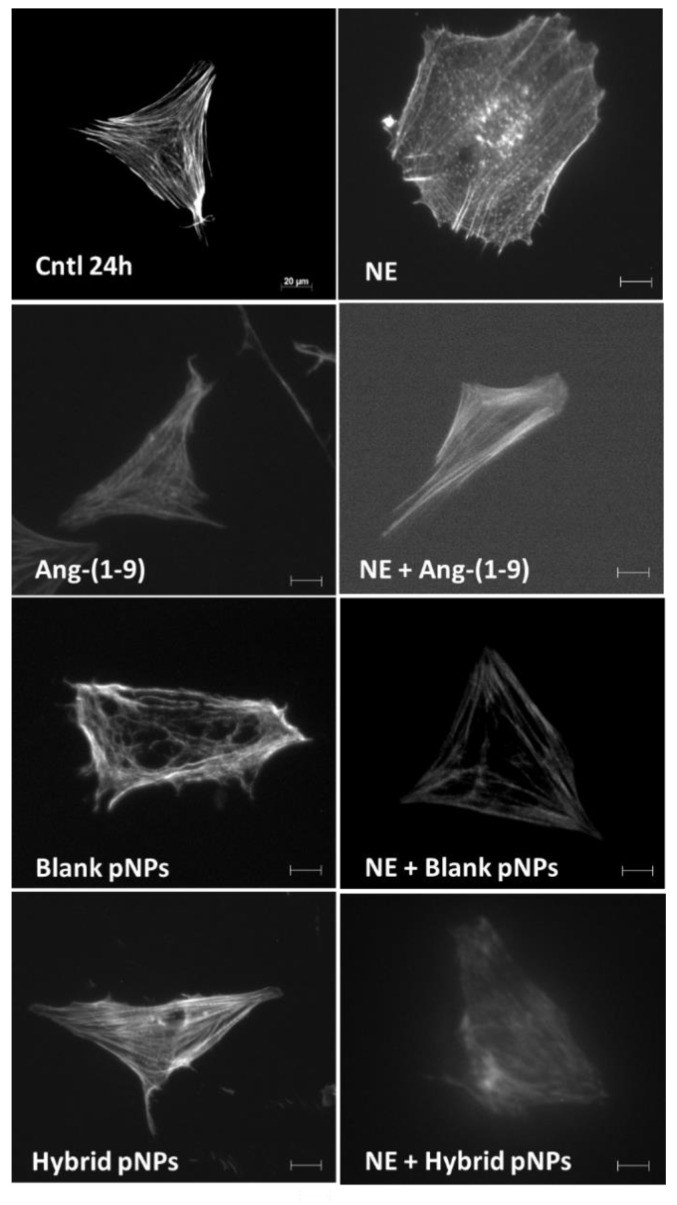
Images of the morphological changes that occur in the neonatal rat cardiomyocyte subjected to the different stimuli. Ctrl 24 h: cardiomyocyte control in MM (maintenance medium) 24 h; NE: cardiomyocytes 24 h + norepinephrine 10 μM; Ang-(1-9): cardiomyocytes 24h + Ang-(1-9) 10 μM; NE + Ang-(1-9): norepinephrine 10 μM + angiotensin-(1-9) 10 μM; NE + Blank pNPs: norepinephrine 10 μM + EE/Alg/Ang-(1-9) pNPs; Hybrid pNPs: cardiomyocytes + EE/Alg/AuNS/Ang-(1-9) pNPs; NE + Hybrid pNPs: norepinephrine 10 μM + pNPs EE/Alg/AuNS/Ang-(1-9). The bar represents 20 µm.

To evaluate the effect of hybrid pNPs on the prevention of the development of NRVM hypertrophy, prior to stimulation with NE, cells were incubated with EE/Alg pNPs, EE/Alg/AuNS 2% + Ang-(1-9) 20% pNPs or Ang-(1-9) not encapsulated, used as a positive control for the prevention of hypertrophy. The area quantification of cardiomyocytes is shown in Figure 9a. The data obtained indicate that while the area of the controls (Ctrl) corresponds to 1716.0 ± 34.7 µm^2^, the treatment with NE increases it to 2170.8 ± 21.4 µm^2^. On the other hand, both treatments with non-encapsulated NE + Ang-(1-9), and with NE + EE/Alg/AuNS 2% + Ang-(1-9) 20% hybrid pNPs significantly decreased the NRVM area compared to control at 1146.4 ± 16.0 and 1103.8 ± 13.9 µm^2^, respectively. On the other hand, although the treatment of NRVM only with blank EE/Alg pNPs in the presence of NE also avoided the increase in the area induced by the hypertrophying agent (1751.7 ± 41.1 µm^2^), the effect on the decrease in area compared to the control, induced by the presence of Ang-(1-9) was not observed. The decrease in the NRVM induced by Ang-(1-9) with respect to the control could be an important factor in the action of Ang-(1-9) to avoid cardiac hypertrophy in vivo; however, future experiments are necessary to answer this hypothesis. Figure 9b shows the graph of the quantification of the perimeter of the NRVM. Our data indicated a significant increase in the perimeter of the NRVM with NE with respect to the CT from 206.4 ± 2.7 to 248.8 ± 2.8 µm. Likewise, the stimuli with NE + Ang-(1-9) or NE + hybrid pNPs reported perimeters of 156.7 ± 2.0 and 154.6 ± 2.3 µm, respectively, showing a significant decrease with respect to the control. Although a downward trend was observed in the perimeter of the NRVM with NE + blank pNPs (166.1 ± 1.7 µm), it was not significant. The images of the morphology of the cardiomyocytes in different treatments are shown in Figure 10.

## 4. Discussion

Physicochemical properties showed that most pNPs have an absolute value of ZP over 20 mV, so they can be classified as moderately stable (±20–30 mV) and highly stable (>±30 mV) [46,47]. The PDI values obtained show that the size dispersion increases when the charge ratio (CR) EE/Alg is higher, although it does not do so in a linear way; however, the values remain below a value of 0.7, which is a limit accepted since heterogeneous particles can cause irregularities in the pharmacokinetic parameters, thus affecting the therapeutic efficacy of a pharmaceutical formulation [48]. Regarding the average size, nano-sized pNPs were obtained and are not affected by CR or by the total number of charges. The results of the incorporation of AuNS in EE/Alg pNPs via the complex coacervation technique indicate an increase in the average hydrodynamic diameter of the EE/Alg/AuNS pNPs with respect to the empty EE/Alg pNPs. In addition, it was found that most of the formulations present positive ZP values. This could be due to the efficient encapsulation by the polymeric material, which predominates over the amount of AuNS. Thus, the positive charge masks the negative ZP of the AuNS. This encapsulation was observed in TEM micrographs.

The encapsulation of Ang-(1-9) in EE/Alg pNPs was shown by DLS; when comparing the size of the empty pNPs with those containing the peptide, it was observed that the EE/Alg/Ang-(1-9) pNPs in which the polyanion predominates present up to <55 nm of difference for the same charge ratios versus empty EE/Alg pNPs. This can be explained in the estimated charge of Ang-(1-9) at pH 4.7 of reaction, which is +2.3 charge units estimated by ProteinCalculator software v3.4 [51]: a positive charge value that would be adding to the positive charges provided by the EE polycation. This would generate a closer proximity between Ang-(1-9) and the polymer chain in the formation process and thus in smaller nanostructures. Regarding ZP, negative values were found in both empty EE/Alg and EE/Alg/Ang-(1-9) pNPs at CR 0.25, in which the alginate polymer predominates and is explained by the contribution of electrical charge negative, generating negative ZP and masking the contribution of positive charge of EE. In contrast, the inversion to a positive ZP from CR 0.5 to 10 was observed in which the contribution of the positive polymer EE is greater in relation to alginate. Consequently, and in general, negative, or positive ZP is dependent on whether negative or positive charges from the polymers at play predominate in the developed NPs.

The determination of the AE% of Ang-(1-9) percentage of the hybrid pNPs for the three formulations evaluated was 63–68%, which shows their high affinity to encapsulate Ang-(1-9). Other authors also reported high AE% of ≈60% heparin in chitosan/hyaluronic acid PE [50]. This characteristic is key and gives the PE complexes an advantage, since they allow the use of low concentrations of polymers to efficiently encapsulate an active principle [51]. Furthermore, it was found that the association efficiency of EE/Alg pNPs by Ang-(1-9) decreases with increasing concentration of Ang-(1-9) incorporated in the formulations. Thus, when observing the AE% of Ang-(1-9) and without considering the percent of incorporation of AuNS that was determined at a fixed value for this study, it is observed that the pNPs EE/Alg/Ang-(1-9) of 2%, 20%, and 40% show a higher AE% when the concentration of incorporated Ang-(1-9) decreases. This same behavior was reported for other nanoparticle systems that encapsulate proteins [52].

On the other hand, the colloidal stability of hybrid EE/Alg/AuNS/Ang-(1-9) pNPs in different relevant media showed a significant change in the hydrodynamic diameter of the hybrid pNPs upon comparing the control with all the media tested at times 0, 15, and 60 min for both temperatures 25 and 37 °C. Furthermore, the largest hydrodynamic diameters were obtained for the case of hybrid pNPs incubated with DMEM and plasma (Figure 5a). This can be attributed to the formation of a protein crown, which is defined as the adsorption of proteins on the surface of NPs [52,53]. This has been demonstrated in other studies with different NPs, media, and cell lines [54,55]. It is precisely this crown of proteins that increases the hydrodynamic diameter of the hybrid pNPs in medium with FBS and plasma, which is a result that is in accordance with that reported by Strojan et al. (2017) [56]. Additionally, the change in size of the hybrid pNPs in DMEM medium with 10% FBS can be attributed to the culture media having sodium chloride (at a concentration of 6 g/L), and when incubated with NPs, an ionic exchange has been described between Ca^2+^ ions and the Na^+^ ions present in the medium, affecting the cross-linking of polymer chains, with an evident increase in their size [57]. As shown in Figure 5b, the PDI values were adequate (close to 0.3) in Milli-Q water, while the PDI of the hybrid pNPs increased significantly for the media in 10% FBS and plasma, which is a consequence of the aggregation that these undergo in biological media due to the neutralization of the surface charge by the ionic species present in the media [58]. In addition, a decrease in the ZP value was observed, see Figure 5c; for the PBS, 10% FBS, and plasma media, the ZP converged to a lower value, which can be explained for the plasma medium due to its composition. The composition consists fundamentally of 90% water and 7% of proteins, of which the most abundant are albumin 45–55% and globulins 5–25%, which present a negative charge depending on the pH of the medium in which they are found. The net charge of albumin at pH 7 is −19. So, at an experimental pH of 4.9, and as this is an acidic environment, more protonable groups are available, and therefore, the charge becomes less negative, which would explain the ZP shift of the hybrid pNPs in plasma.

In vitro release studies of Ang-(1-9) indicate that the release of Ang-(1-9) from EE/Alg pNPs with CR 0.5 and 10 was 4% and 12% after the first hour, and after 48 h, it reached a maximum release of 20 and 25%, respectively. This profile shows that during the study time, a complete release of the content of Ang-(1-9) contained in the pNPs was not achieved. To understand the release pattern, several factors must be considered, such as the amount of Ang-(1-9) charged, the ratio of EE/Alg polymers in the evaluated CR, the properties of the polymers used (size of the chain of the polymer, flexibility, mobility, water adsorption), as well as the interactions between the polymers and the encapsulated Ang-(1-9). The fact of not seeing the release of the peptide that is efficiently associated with the nanoparticle in the total study time of 48 h may be supported by what was reported by Balmert et al. (2015) [59], who demonstrated that electrostatic interactions are established between a positively charged peptide which reacts with the negatively charged polymer and thus restricts diffusion of the peptide through the matrix. With this, a slow-release kinetics is generated from the nanoparticle. Both EE and Alg are high molecular weight polymers, and their solutions in water are viscous [60]. This high viscosity could be favouring the encapsulation process of the peptide and therefore a much more sustained release over time. Consistent with this, the viscosity of the alginate solution has been shown to increase as the pH decreases [61], which for the purposes of this test was at pH 7.4. This results in a high viscosity solution, which would form a strong wall containment nanoparticle encapsulating Ang-(1-9) and thus a lower hydration capacity of the polymeric nanostructure and thus a slower release profile.

The release profile of Ang-(1-9) from EE/Alg/AuNS 2 and 20% pNPs showed that a burst release of Ang-(1-9) occurs after 15 min; this means that a high percentage of the initial amount is released—in this case, 62.9 ± 4.0% and 75.1 ± 8.1% for AuNS 2 and 20%, respectively. After this initial release, complete release is achieved after 120 and 105 min, respectively. This phenomenon has been previously reported by other authors and can be analyzed from two perspectives: it is often considered as a negative consequence of the creation of long-term controlled release systems, while alternatively, a rapid release or high-rate initial administration may be desirable [62]. In the case of Ang-(1-9) release, this could be an advantage as it allows an initial loading of the peptide and then a controlled release. The cause of this burst release has been attributed to a variety of physical, chemical, formulation parameters, surface characteristics, and triggering mechanisms, to name a few [62]. However, for this test, it is believed that it could be due to the contribution by a part of the free Ang-(1-9) bound to the surface of the hybrid pNPs, which is released rapidly once in the middle of release: a phenomenon that has been observed and described by other authors. In addition, there is a contribution because of temperature, as previously reported by Gutowska et al. (1995), who developed N-isopropylacrylamide hydrogels for the release of heparin, showing that once in the release medium at 37 °C, there was burst release, followed by release slower than initial [63]. After the burst release has elapsed, a gradual and very similar release kinetics is observed until 100% release is completed.

In vitro safety results of NRVM culture showed that Ang-(1-9) does not affect the viability of cardiomyocytes treated at different times (24, 48, and 72 h) with Ang-(1-9) at concentrations of 1, 10, 100 nM, and 1 μM. The exposure to Ang-(1-9) does not produce changes in cardiomyocyte viability at any of the concentrations or exposure times evaluated. The cell viability assays did not show significant differences at the different concentrations evaluated of pNPs.

In vitro results of anti-hypertrophic efficacy of hybrid EE/Alg/AuNS 2% + Ang-(1-9) 20% pNPs on NRVM subjected to stimuli with norepinephrine (NE) showed that the area and perimeter quantification of cardiomyocytes suggest that the encapsulation of Ang-(1-9) reports similar results to those obtained with non-encapsulated Ang-(1-9), indicating that our formulation of hybrid EE/Alg/AuNS 2% + Ang-(1-9) 20% pNPs is effective in preventing cardiomyocyte hypertrophy as they can release enough of the Ang-(1-9) peptide to cause the effect.

Finally, the osmolarity of the EE/Alg pNPs and EE/Alg/AuNS 2% + Ang-1-9) 20% pNPs solutions and the maintenance medium of the primary cardiomyocyte cultures were calculated, obtaining an osmolarity of 300 mosmol/L for all cases. Therefore, the cardiac cells were not physiologically exposed to large fluctuations in external osmolarity. All these results together show the potential of these new hybrid EE/Alg/AuNS pNPs for use in biomedical applications and support the projections for the development of nanocarriers implemented by coacervation for the delivery of biologicals. Furthermore, and considering the uses assigned to nanoparticles for the diagnosis and monitoring of treatment in various pathologies, the use of these hybrid pNPs could open a theragnostic field in heart disease.

## 5. Conclusions

In this work, it was possible to obtain a hybrid carrier (EE/Alg/AuNS pNPs) based on the complex coacervation of anionic alginate and cationic polymethacrylate polyelectrolytes with the incorporation of gold nanospheres for the supply of the anti-cardiac hypertrophic peptide Ang-(1-9). Incubation of the hybrid pNPs in different media changed the physicochemical parameters of the pNPs. Furthermore, the results suggest that the incubation of the hybrid pNPs in biological media such as FBS and plasma can lead to the adsorption of proteins from the medium on the surface of the nanoparticles, which determines the formation of a protein crown. The incorporation of AuNS did not cause negative effects on cytotoxicity, nor did it affect the association capacity of Ang-(1-9), so this hybrid carrier EE/Alg/AuNS pNPs could be used as an efficient delivery carrier for the Ang-(1-9) peptide, having the additional advantage of being able to be monitored by imaging techniques, thus showing potential applications in theragnostic. In the release kinetics of the Ang-(1-9) peptide from hybrid EE/Alg pNPs with AuNS, it was observed that a burst release of Ang-(1-9) occurs after 15 min; this means that a high percentage of the initial amount is released, in this case 62.9 ± 4.0% and 75. 1 ± 8.1% for AuNS 2 and 20%, respectively. We believed that it could be due to the contribution by a part of the free Ang-(1-9) bound to the surface of the hybrid pNPs, which is released rapidly once in the middle of release. Efficacy studies in cardiomyocytes demonstrated that the application of hybrid pNPs of EE/Alg/AuNS 2% + Ang-(1-9) 20% as pre-treatment in neonatal rat cardiomyocytes and subsequent incubation with NE was able to prevent the development of NRVM hypertrophy. Cardiac hypertrophy is a chronic process and therefore may require high concentrations and constant exposure to the peptide Ang-(1-9) to counteract pro-growth signals, so a conventional treatment with Ang-(1-9) is impractical due to its short half-life, as it is constantly degraded by ACE. Based on our results, this new delivery system for Ang-(1-9) has the potential to effectively prevent the development of cardiac hypertrophy.

## Figures and Tables

**Table 1:** Effect of the relationship between positive and negative charge in the manufacturing process of pNPs. Results for size, PDI, and ZP. Data expressed as mean ± SD (*n* = 3).

Formulation		Size (nm)	PDI	ZP (mV)
TC ^1^	CR ^1^ [EE/Alg]			
15	0.1	101.3 ± 1.49	0.265 ± 0.007	−36.8 ± 2.5
15	0.25	150.1 ± 0.9	0.112 ± 0.001	−33.2 ± 0.6
15	0.5	150.0 ± 1.1	0.149 ± 0.008	40.0 ± 1.1
15	0.75	107.8 ± 0.3	0.291 ± 0.034	37.8 ± 2.6
15	1	93.5 ± 1.8	0.283 ± 0.023	36.1 ± 3.0
15	1.33	95.9 ± 1.1	0.216 ± 0.007	27.5 ± 13.6
15	2	82.4 ± 1.3	0.330 ± 0.045	62.6 ± 12.7
15	4	71.5 ± 0.9	0.366 ± 0.012	87.6 ± 9.5
15	10	123.7± 1.4	0.339 ± 0.046	30.9 ± 4.7

^1^ TC = total charge; CR = charge ratio.

**Table 2:** Values of size, PDI, and ZP obtained for the empty EE/Alg pNPs and EE/Alg/AuNS pNPs. Data are expressed as mean ± SD (*n* = 3).

Formulation		Size (nm)		PDI		ZP (mV)	
TC ^1^	CR ^1^	EE/Alg	EE/Alg/AuNS	EE/Alg	EE/Alg/AuNS	EE/Alg	EE/Alg/AuNS
15	0.25	150.1 ± 0.9	119.0 ± 0.6	0.112 ± 0.001	0.155 ± 0.013	−33.2 ± 0.6	−35.6 ± 1.2
15	0.5	150.0 ± 1.1	301.8 ± 11.0	0.149 ± 0.008	0.325 ± 0.014	40.0 ± 1.1	22.3 ± 1.5
15	0.75	107.8 ± 0.3	140.9 ± 0.5	0.291 ± 0.034	0.160 ± 0.009	37.8 ± 2.6	32.8 ± 6.1
15	1	93.5 ± 1.8	132.1 ± 3.0	0.283 ± 0.023	0.233 ± 0.013	36.1 ± 3.0	41.1 ± 1.4
15	1.33	95.9 ± 1.1	116.4 ± 1.7	0.216 ± 0.007	0.196 ± 0.021	27.5 ± 13.6	35.2 ± 3.2
15	2	82.4 ± 1.3	110.3 ± 0.8	0.330 ± 0.045	0.154 ± 0.012	62.6 ± 12.7	34.0 ± 4.3
15	4	71.5 ± 0.9	101.5 ± 0.9	0.366 ± 0.012	0.233 ± 0.044	87.6 ± 9.5	34.3 ± 1.0
15	10	123.7± 1.4	117.2 ± 9.4	0.339 ± 0.046	0.391 ± 0.015	30.9 ± 4.7	23.0 ± 4.3

^1^ TC = total charge; CR = charge ratio.

**Table 3:** Values of size, PDI, and ZP obtained for the empty EE/Alg pNPs and EE/Alg/Ang-(1-9) pNPs. Data expressed as mean ± SD (*n* = 3).

Formulation			Size (nm)		PDI		ZP (mV)	
TC ^1^	CR ^1^	[Ang-(1-9)] (µg/µL)	EE/Alg	EE/Alg/ Ang-(1-9)	EE/Alg	EE/Alg/ Ang-(1-9)	EE/Alg	EE/Alg/ Ang-(1-9)
15	0.25	0.0092	150.1 ± 0.9	107.0 ± 5.7	0.112 ± 0.001	0.172 ± 0.021	−33.2 ± 0.6	−36.0 ± 1.9
15	0.5	0.0096	150.0 ± 1.1	108.5 ± 3.7	0.149 ± 0.008	0.205 ± 0.020	40.0 ± 1.1	39.9 ± 5.5
15	0.75	0.0099	107.8 ± 0.3	116.3 ± 7.6	0.291 ± 0.034	0.186 ± 0.015	37.8 ± 2.6	38.2 ± 0.2
15	1	0.0102	93.5 ± 1.8	130.0 ± 3.4	0.283 ± 0.023	0.153 ± 0.031	36.1 ± 3.0	33.1 ± 0.1
15	1.33	0.0104	95.9 ± 1.1	142.2 ± 1.1	0.216 ± 0.007	0.130 ± 0.021	27.5 ± 13.6	39.0 ± 0.3
15	2	0.0107	82.4 ± 1.3	113.7 ± 1.1	0.330 ± 0.045	0.150 ± 0.016	62.6 ± 12.7	35.7 ± 0.7
15	4	0.0112	71.5 ± 0.9	101.5 ± 0.9	0.366 ± 0.012	0.178 ± 0.012	87.6 ± 9.5	30.7 ± 0.9
15	10	0.0116	123.7± 1.4	121.8 ± 0.4	0.339 ± 0.046	0.242 ± 0.005	30.9 ± 4.7	26.3 ± 2.2

^1^ TC = total charge; CR = charge ratio.

**Table 4:** Values of size, PDI, and ZP obtained for the EE/Alg/AuNS 2% + Ang-(1-9) 2%, 20% and 40% pNPs. Data expressed as mean ± SD (*n* = 3).

Formulation	TC ^1^	CR ^1^	Size (nm)	PDI	ZP (mV)
EE/Alg/AuNS 2% + Ang-(1-9) 2%	15	0.5	108.1 ± 2.4	0.203 ± 0.0	−35.1 ± 0.2
	15	10	98.0 ± 1.1	0.231 ± 3.7	38.0 ± 0.01
EE/Alg/AuNS 2% + Ang-(1-9) 20%	15	0.5	98.1 ± 4.4	0.220 ± 0.2	−34.8 ± 0.7
	15	10	86.1 ± 1.1	0.251 ± 0.0	37.3 ± 2.5
EE/Alg/AuNS 2% + Ang-(1-9) 40%	15	0.5	85.9 ± 1.1	0.404 ± 1.1	−39.4 ± 2.5
	15	10	86.3 ± 1.3	0.450 ± 1.7	46.6 ± 3.5

^1^ TC = total charge; CR = charge ratio.

## Supplementary Materials

The following are available online at https://www.mdpi.com/article/10.3390/pharmaceutics13060822/s1, Figure S1: AuNS physicochemical characterization, Figure S2: Cytotoxicity of Ang-(1-9) in primary culture of cardiomyocyte by flow cytometry, Figure S3: Cellular viability essays: MTS.
