# Supplementary material for: Nanoparticle-Mediated Angiotensin-(1-9) Drug Delivery for the Treatment of Cardiac Hypertrophy
Source: Pharmaceutics. 2021 Jun 1;13(6):822. doi: 10.3390/pharmaceutics13060822 (PMC8228229; doi:10.3390/pharmaceutics13060822)
Supplement: Supplementary file 1 [file pharmaceutics-13-00822-s001.zip › pharmaceutics-1218230-supplementary.pdf]

pharmaceutics

MDPI

Supplementary Materials: Nanoparticle-Mediated Angiotensin-(1-9) Drug Delivery for the Treatment of Cardiac Hypertrophy

Sabrina Sepúlveda-Rivas, Matías S. Leal, Zully Pedrozo, Marcelo J. Kogan, María Paz Ocaranza and Javier O. Morales

1. AuNS Physicochemical Characterization

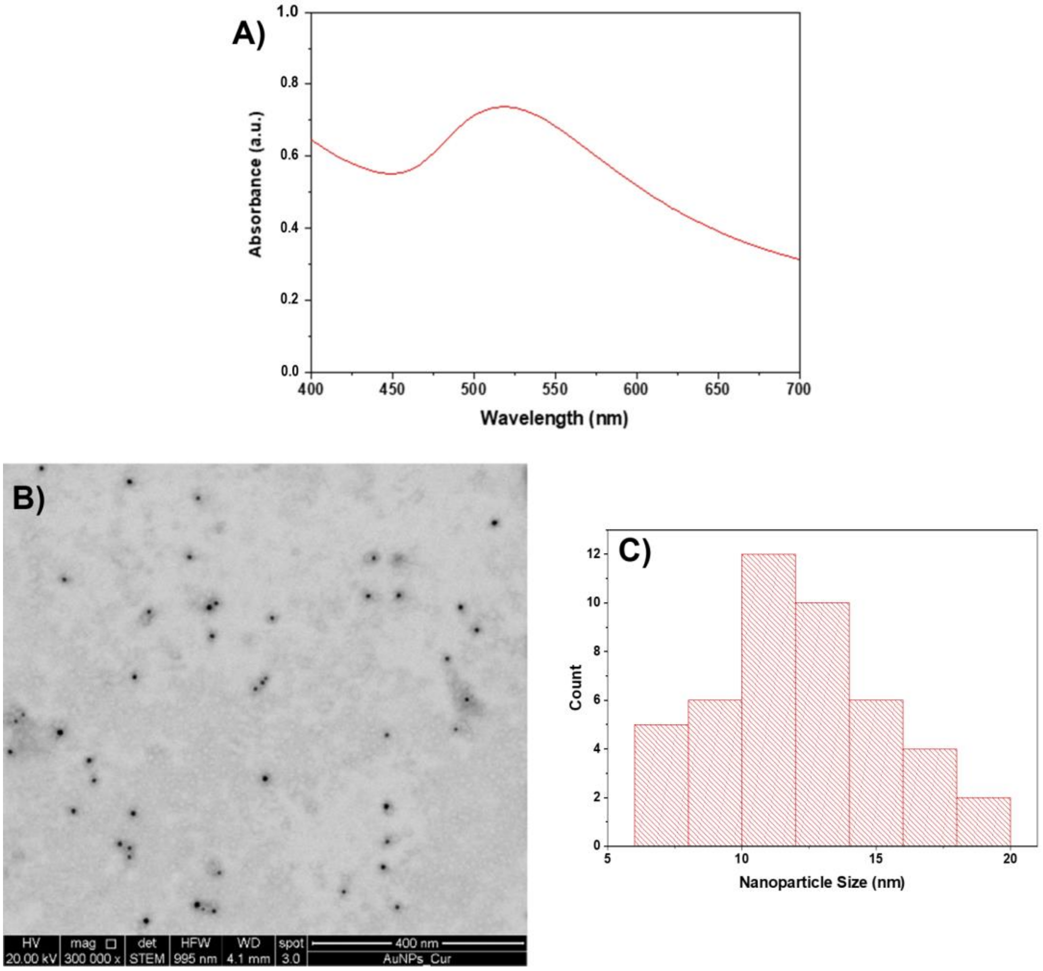

Figure S1. Characterization of the AuNS. (**A**) UV-Vis absorption spectrum of AuNS; (**B**) TEM photomicrograph of AuNS with an (**C**) insert of its size distribution.

2. Cytotoxicity of Ang-(1-9) in Primary Culture of Cardiomyocyte by Flow Cytometry

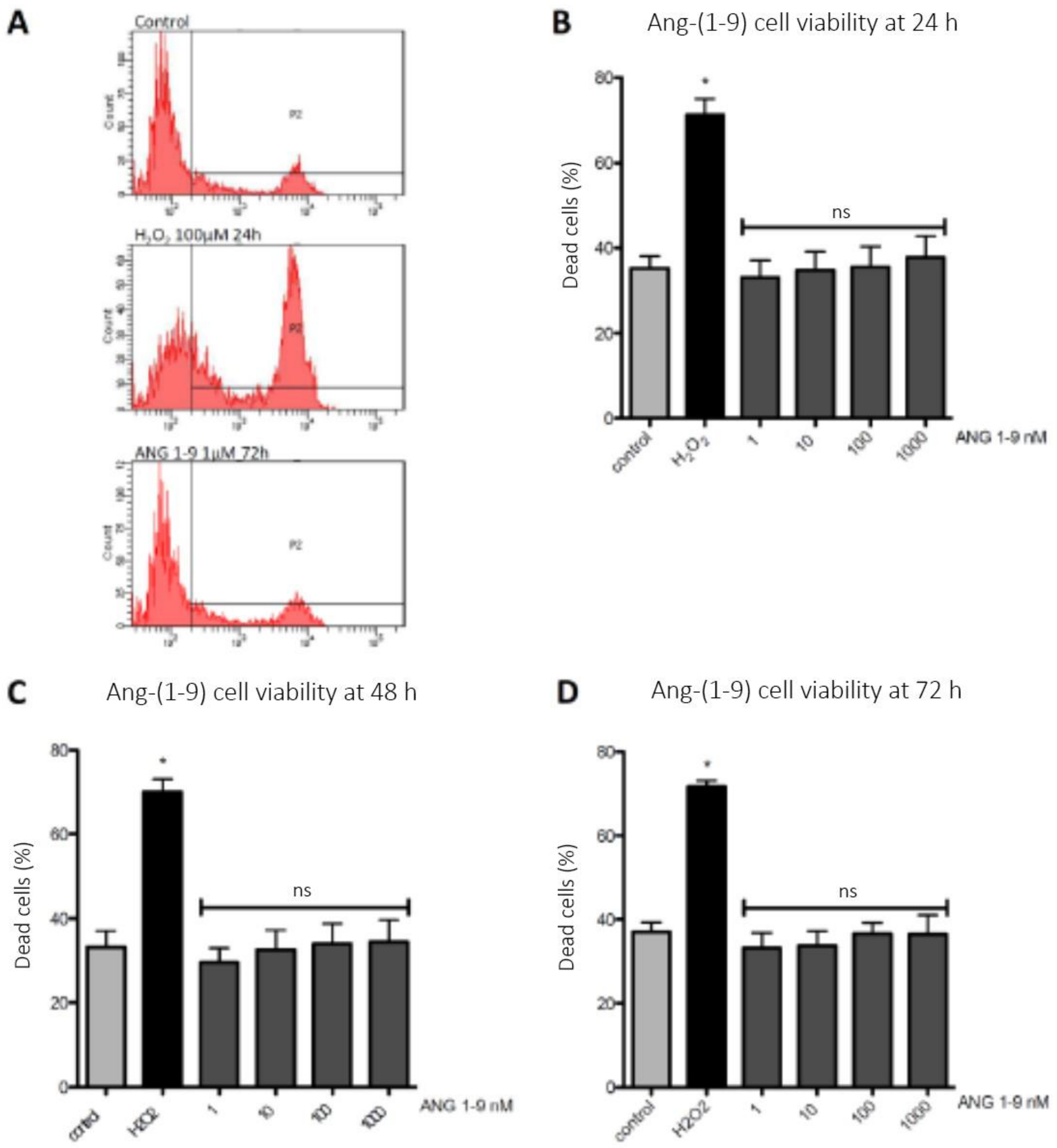

Figure S2. **(A)** Representative histograms of PI incorporation measurement by flow cytometry. The viability of cardiomyocytes incubated with Ang-(1-9) at concentrations 0, 1, 10, 100 nM, and 1 μM was determined, during **(B)** 24 h, **(C)** 48 h, and **(D)** 72 h, the data correspond to the mean ± SEM of five independent experiments. One-way ANOVA analysis, with Dunnett's post-test. * $p < 0.05$ ns = not significant.

3. Cellular Viability Essays: MTS

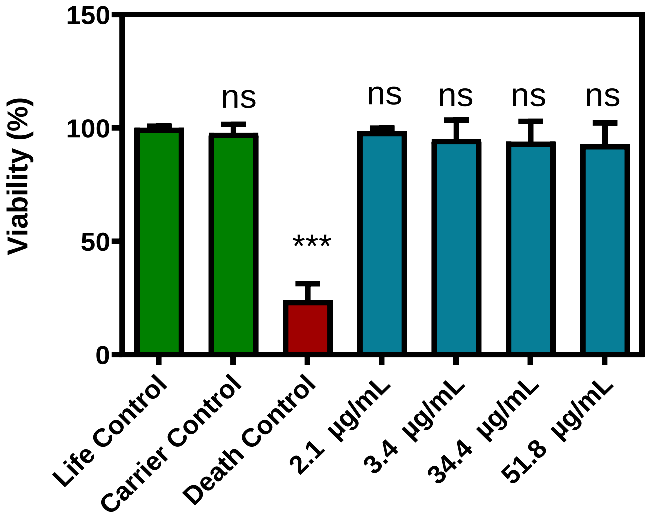

Figure S3. Viability expressed as a percentage of cell viability of neonatal rat cardiomyocytes treated with different concentrations of EE/Alg pNPs compared to the culture medium control. Life control: DMEM/10% FBS, cell death: 10% SDS, vehicle: Milli-Q water, gold nanospheres: 2% AuNS and kept at 37 °C and 5% CO_2 for 1 h. The data represent mean values ± SD (n = 3), ***pP < 0.001, ns = not statistically significant with respect to the life control of ANOVA-Dunnett's.
